# Cytophysiological manifestations of wheat’s defense reactions against stem rust induced by the biofungicide Novochizol

**DOI:** 10.18699/vjgb-25-57

**Published:** 2025-07

**Authors:** A.B. Shcherban, L.Ya. Plotnikova, V.V. Knaub, E.S. Skolotneva, V.V. Fomenko

**Affiliations:** Kurchatov Genomic Center of ICG SB RAS, Novosibirsk, Russia Institute of Cytology and Genetics of the Siberian Branch of the Russian Academy of Sciences, Novosibirsk, Russia; Omsk State Agrarian University named after P.A. Stolypin, Omsk, Russia; Omsk State Agrarian University named after P.A. Stolypin, Omsk, Russia; Kurchatov Genomic Center of ICG SB RAS, Novosibirsk, Russia Institute of Cytology and Genetics of the Siberian Branch of the Russian Academy of Sciences, Novosibirsk, Russia; N.N. Vorozhtsov Novosibirsk Institute of Organic Chemistry of the Siberian Branch of the Russian Academy of Sciences, Novosibirsk, Russia

**Keywords:** biopesticides, Novochizol, common wheat, stem rust, resistance mechanisms, ROS, phenols, биопестициды, Новохизоль, мягкая пшеница, стеблевая ржавчина, механизмы устойчивости, АФК, фенолы

## Abstract

Biologization is a priority direction of agricultural production. One of the promising approaches to solve the biologization problem is the use of chitosan-based biopreparations to stimulate plant growth and protect plants from a wide range of pathogens. Currently, active work is underway to create and test new chitosan preparations. Novochizol was obtained as a result of intramolecular crosslinking of linear chitosan molecules and has a globular shape. Previously, a Novochizol-stimulating effect on the growth and development of common wheat was demonstrated. However, the induced resistance mechanisms against rust diseases have not been studied before. The reported studies have revealed the dose effect of the preparation on the development of wheat stem rust. The best results of visual estimation of plant reactions were obtained with 0.125 and 0.75 % Novochizol pretreatment four days before rust infection. After pretreatment of susceptible cv. Novosibirsk 29 seedlings, a resistant reaction appeared and the urediniopustule density was decreased. Cytophysiological studies have shown that 0.75 % Novochizol stimulated an intensive accumulation of hydrogen peroxide Н2О2 in the leaves of the infected and healthy plants within 48 hours post inoculation (h p/in). During the period of 48–144 h p/in, H2O2 gradually disappeared from tissues, but its content increased significantly at the sporulation stage around pustules. However, Novochizol did not induce the hypersensitivity reaction in infected plants. The preparation induced an earlier and more intensive (compared with untreated plants) accumulation of phenolic substances with different autofluorescence in the zones around pathogen colonies. Novochizol induced a change in the ratio of phenols with different spectral characteristics towards compounds with an increased content of syringin derivatives. This work is the first stage in the study of Novochizol effects on wheat defense mechanisms against stem rust. The research will be continued using molecular genetics, biochemical and cytophysiological methods.

## Abstract (ru)

Биологизация земледелия считается приоритетным направлением сельскохозяйственного производства. Одним из перспективных подходов к решению задачи биологизации является применение препаратов на основе хитозана для стимуляции роста и защиты растений от широкого круга патогенов. В настоящее время проводятся активные работы по созданию и испытанию новых форм хитозановых препаратов. Препарат «Новохизоль» получен в результате внутримолекулярных сшивок линейных молекул хитозана и имеет глобулярную форму. Ранее установлено стимулирующее влияние Новохизоля на рост и развитие мягкой пшеницы, однако индуцируемые защитные механизмы против ржавчинных болезней не изучались. Проведенные исследования показали дозовый эффект препарата на развитие стеблевой ржавчины пшеницы. При обработке за четверо суток до заражения лучшие результаты по развитию устойчивой реакции растений, сокращению числа и размеров пустул были получены с Новохизолем в концентрации 0.125 и 0.75 %. После предобработки на проростках восприимчивого сорта Новосибирская 29 проявилась устойчивая реакция и снизилось число пустул. Цитофизиологические исследования показали, что обработка 0.75 % Новохизолем стимулировала интенсивное накопление пероксида водорода Н2О2 в листьях инфицированных и здоровых растений в течение 48 ч после инокуляции. В период 48–144 ч после инокуляции Н2О2 постепенно исчезал из тканей, но на стадии спороношения его содержание значительно возрастало в зоне колоний и пустул. Новохизоль не индуцировал развитие реакции сверхчувствительности в зараженных растениях. Применение препарата способствовало более раннему и интенсивному (по сравнению с необработанными растениями) накоплению фенольных веществ с разным спектром автофлуоресценции в зоне колоний патогена. Препарат повлиял на изменение соотношения фенолов с разными спектральными характеристиками в сторону соединений с повышенным содержанием остатков сирингина. Данная работа является первым этапом изучения действия Новохизоля на защитные механизмы пшеницы против стеблевой ржавчины. Исследования будут продолжены с применением молекулярно-генетических и биохимических методов.

## Introduction

Due to the proposed rise in the world’s population to 9.5 billion people by 2050, it is necessary to increase grain production by 1.7 times (USDA, 2016). An increase in wheat grain harvests can be achieved by breeding more productive and stress-resistant varieties, as well as reducing losses caused by abiotic and biotic factors. Synthetic pesticides are traditionally widely used to protect crops from diseases and pests. These protective agents are highly effective; however, they can be accumulated in plants and soils, having a negative effect on the ecological situation in agrocenoses and product quality (Sternshis et al., 2016). The use of biological pest management agents (BPMA) increases stress resistance mechanisms (Chandler et al., 2011).

BPMA based on natural compounds and beneficial microorganisms attract the attention of researchers and practitioners. These substances are often close to chemical pesticides in effectiveness, but do not have their disadvantages (Chakraborty et al., 2020). The range of biopesticides and their application schemes are very diverse, which is determined by the pathogens and pests’ biology, as well as their interaction with plants. BPMA may inhibit the pathogens and pests directly or induce a complex of plant resistance reactions (Orzali et al., 2017; Yarullina et al., 2023).

Chitin and chitosan derivatives are widely used as BPMA (Tyuterev, 2015; Malerba, Cerana, 2016). Polymer carbohydrate chitin is widespread in nature, as components of integuments of arthropods (including crustaceans and insects) and fungi. Chitosan is produced by chitin hydrolysis and deacetylation. Chitosan-based preparations have a stimulating effect on plant growth and development, as well as enhance resistance to abiotic stresses (Haggag et al., 2014; Orzali et al., 2017). Chitosan derivatives are also of particular interest as inducers of resistance to fungal, bacterial, and viral diseases (Chakraborty et al., 2020; Shcherban, 2023).

Chitosan preparations may differ in their main characteristics: molecular weight, deacetylation degree, and polydispersity index (Richter et al., 2012). The effectiveness of chitosan derivatives can be significantly enhanced by their modification, such as the introduction of functional groups of Schiff bases, halogen atoms (Cl or F), metal nanoparticles, urea groups, etc. (Varlamov et al., 2020; Yarullina et al., 2023). Preparations based on conjugates of chitosan with phenolic hydroxycinnamic acids (ferulic and caffeic) have proven promising for protecting plants from fungal and viral diseases (Rkhaila et al., 2021; Yarullina et al., 2024a). A positive effect of combining chitosan preparations with other biologically active substances and beneficial microorganisms (Plant Growth Promoting Bacteria, PGPB) has been established (Rkhaila et al., 2021; Yarullina et al., 2024b). The protective effects strengthening is due to the synergistic action of different drug components (Tyuterev, 2015). Currently, a wide range of chitosan-based BPMA have been created in the world and their tests have been carried out on various cultures. A comparison of the results showed that their stimulating and protective effects depended on preparation compositions, as well as plant and pathogen species (Rabea et al., 2005; Orzali et al., 2017).

A number of complex chitosan preparations with the addition of biologically active substances have been developed in Russia, including “Narcissus” with succinic and glutamic acids; “Chitosar M” with salicylic acid (SA); “Chitosar F” with arachidonic acid; an agent with SA and vanillin, etc. (Tyuterev, 2015; Popova et al., 2018). The combined agents were effective against different pathogenic fungi, viruses and pests. Their application enhanced crop resistance to diseases, such as that of wheat to leaf rust, spot blotch and root rot; rice, to Pyricularia; tomatoes, to late blight and Fusarium fruit rot; potatoes, to late blight and Y virus; cucumbers, to downy mildew, etc. (Tyuterev, 2015; Badanova et al., 2016; Popova et al., 2018).

A promising new chitosan derivative is “Novochizol”, obtained by intramolecular crosslinking of linear chitosan molecules. Novochizol has a globular shape, which gives it a number of advantages over chitosan, namely increased solubility in aqueous solutions, chemical stability, resistance to biodegradation, high adhesion and ability to penetrate tissues. This form is able to absorb various substances and slowly release them into plants after application (Novochizol SA, www.novochizol.ch). These properties are important for creating promising combined agents with other biologically active substances. Novochizol has a growth-stimulating effect when processing seeds and leaves. It was shown that this substance enhanced common wheat seed germination, contributed to an increase in root and total plant weight (Teplyakova et al., 2022). The effectiveness of complex Novochizol preparations with usnic acid or Siberian pine bark extract for protecting wheat from root rot and Septoria blotch was proved in the field (Burlakova et al., 2025).

It is known that after plant recognition of non-specialized or avirulent pathogen effectors (elicitors), a set of defence reactions is activated. The earliest responses include the reactive oxygen species (ROS) and nitric oxide NO generation (Manjunatha et al., 2009; Singh et al., 2021; Plotnikova, Knaub, 2024). ROS ( O •2–, H2O2, •OH, 1O2) accumulation leads to a splash of oxidative reactions, called an oxidative burst. The enzyme superoxide dismutase (SOD) converts the superoxide anion O •2– into the hydrogen peroxide H2O2 (Maksimov, Cherepanova, 2006). H2O2 has a toxic effect on pathogens, and is a messenger in NADP·H-oxidase signaling system implemented through a SA-dependent signaling cascade (Tarchevsky, 2000; Yarullina et al., 2023). As a result of SA-dependent cascade action, a complex of resistance mechanisms against biotrophic pathogens is implemented in the infection zone, including ROS generation, hypersensitive reaction (HR), defence PR proteins (Pathogenesis-Related Proteins) and phenolic substances synthesis. Defence reactions against necrotrophic pathogens are realized using a signaling cascade dependent on jasmonic acid (JA), abscisic acid and ethylene. The resistance to hemibiotrophs is ensured by the combined action of the SA- and JA-dependent cascades (Singh et al., 2021; Yarullina et al., 2023). The study of the chitosans’ effects on defence reactions showed activation of the ROS and phenolic metabolism enzymes, PR proteins accumulation and cell wall strengthening with the lignin and callose (Orzali et al., 2017; Shcherban, 2023).

To develop BPMA technology, it is necessary to learn their effect on defence mechanisms and the development of the most devastating diseases. Novochizol action on wheat resistance mechanisms against rust diseases has not been studied before. The aim of the work was to study the Novochizol effect on the defence mechanisms of a susceptible common wheat variety infected with the stem rust fungus Puccinia graminis f. sp. tritici Erikss. et Henn.

## Materials and methods

Plant material. The objects of the research were 10-day-old seedlings of the spring common wheat cv. Novosibirskaya 29 susceptible to stem rust. Plants were grown in pots with soil as recommended for experiments with rust fungi by international protocols (Woldeab et al., 2017). The seedlings were treated with Novochizol solutions at concentrations of 0.125, 0.75, 1.5, and 2.5 %. Solutions were applied to plants (15 ml per 100 plants) using a sprayer four days before infection with stem rust. Such a pretreatment period is sufficient to induce defensive effects by BPMA, including chitosan derivatives, against oomycetes and rust fungi (Faoro et al., 2008; Bellameche et al., 2021; Elsharkawy et al., 2022). Plants treated with bidistilled water served as a control.

The seedlings were inoculated with urediniospores of a mixed sample of the West Siberian population of P. graminis f. sp. tritici (Pgt), included isolates with avirulence/ virulence genes to wheat genes Sr11Sr24Sr30Sr31/Sr5Sr9aSr9b Sr9dSr9gSr10Sr17Sr38SrMcN. The urediniospores were stored at –70 °C before the experiment and revitalized using susceptible common wheat cv. Khakasskaya (Rsaliyev A.S., Rsaliyev Sh.S., 2018). Urediniospore suspension at the concentration of 0.8 mg/ml Novec 7100 (Sørensen et al., 2016) was applied to seedlings using a sprayer. Inoculated plants were incubated for 24 h in a humid chamber in the dark at a temperature of 15–20 °C for maximal spore germination. After that, the plants were transferred to growth chambers and incubated under 16 h illumination with an intensity of 10,000 lux at a temperature of 26–28 °C. Such temperature is critical for full appressoria structure formation and pathogen penetration into the stomata, and infection hyphae development in the plant tissue (Roelfs et al., 1992).

 Phytopathologicalassessment of plant reaction to infection. The effect of Novochizol was assessed by quantitative and qualitative characteristics used to describe the resistance of wheat seedlings to stem rust, such as pustule density (number per leaf, 10 plants per variant) and reaction type. Plant reaction (infection type, IT) was determined 12–14 days post inoculation (p/in) using a modified Stackman scale. The ITs “0”, “;”, “1”, and “2” were interpreted as resistant (R), and “3”, “3+” and “4”, as susceptible (S) (Roelfs et al., 1992).

Cytological and cytochemical methods. The studies were carried with plants treated with 0.75 % Novochizol. The material was fixed at 0, 24, 96, 144 and 240 h p/in in lactophenol fixative (phenol, lactic acid, glycerin, distilled water, 96 % ethanol, in the ratio of 1 : 1 : 1 : 1 : 8) (Plotnikova, Meshkova, 2009). Infection structures on the surface and in plant tissues were detected using the fluorescent dye Uvitex 2B (Sigma- Aldrich, USA) by a modified method (Moldenhauer et al., 2006). For this, the material fixed in lactophenol was washed with distilled H2O, and afterwards it was kept for 3 h in acetic alcohol (96 % ethanol and glacial acetic acid, in the ratio of 3 : 1). After washing with distilled H2O, the leaf pieces were kept in a series of liquids, such as 50 % ethanol (20 min), 0.5N NaOH (30 min), distilled H2O (5 min), 0.1M Tris-HCl buffer pH 5.8 (30 min), and distilled H2O (5 min). Staining was carried out for 15 minutes in 0.1 % Uvitex 2B in 0.1M Tris-HCl (pH 5.8), preheated at 60 °C. To differentiate the colour, the material was kept in distilled water for 90 min. The observations were carried out in reflected light with an excitation wave of λmax = 355 nm and an emission wave of λmax = 420 nm. Undamaged fungal structures showed a blue fluorescence, the damaged plant cells and pathogen hyphae were light blue or white.

For hydrogen peroxide H2O2 localization in tissues, a vital staining of the material with 0.02 % 3,3′-diaminobenzidine tetrachloride (DAB, Sigma-Aldrich, USA) was implemented before fixation (Plotnikova, Meshkova, 2009). The DAB solution was infused into the leaves by vacuum infiltration and incubated for 30 min. Insoluble cherry formazane was formed in the presence of H2O2.

Phenolic substances distribution in the leaves was studied using special reaction with aniline sulfate to common phenols (low molecular weight phenols and polymer lignin). The material was stained with 1 % aniline sulfate (aniline sulfate, glacial acetic acid, and 50 % ethyl alcohol, in the ratio of 1 : 2 : 97) for 1 h, followed by washing in distilled water (Japaridze, 1953). The lignins in the veins and plant cell walls in the infection zones were coloured yellow-brown. Additionally, the phenols autofluorescence in reflected light with an excitation wave of λmax = 355 nm and emission of λmax = 530 nm (green fluorescence) or λmax = 605 nm (red fluorescence) was studied (Plotnikova, Meshkova, 2009). Cytological studies were carried out using an ARSTEK E62 light microscope (ARSTEK, China) with a Sony Alpha A6400 APS-C digital camera (the resolution of 24.2 MP/inch, Sony, Japan).

The results of 30–50 vbgPgt urediniospores development in each of the five plants per variant were studied at each experiment stage, and were counted as repetitions. The areas of mycelium and urediniopustule (35–50 pcs. per variant) were measured after 240 h p/in, using the camera software. The mean values and standard errors were determined (in tables and graphs), and the least significant difference at p ≤ 0.05 (LSD0.05) was calculated.

## Results

**Visual assessment of the Novochizol effect on stem rust development**

At the first stage of the work, the effect of different Novochizol concentrations on the disease development in the susceptible cv. Novosibirskaya 29 seedlings was studied. A wide range of preparation concentrations was used in the experiments, from 0.125 to 2.5 %. The pustules with IT “4” were formed on plants treated with water (control). Any Novochizol concentration influenced the disease development. It could be seen in the decrease of pustule density, pustule size reduction, and chlorosis appearance around the pustules (Table 1). IT decreased to the least extent when 2.5 % Novochizol solution was used (IT “3”, “3+”). The treatments with 0.125, 0.75 and 1.5 % concentrations induced resistant reactions. The pustules sizes decreased to the greatest extent when the plants were treated with 0.75 % Novochizol (IT “2”, “2–”). This experimental variant was used for studying plant defence reactions.

**Table 1.**
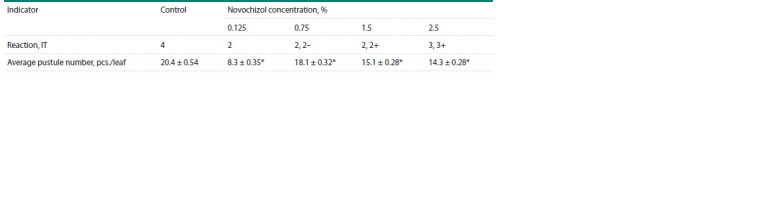
Results of a visual assessment of the Novochizol concentration effect on the development of P. graminis f. sp. tritici in wheat seedlings * Significant differences with the control at р ≤ 0.05.

**Results of cytophysiological studies of the Novochizol effect on pathogenesis**

**Fig. 1.**
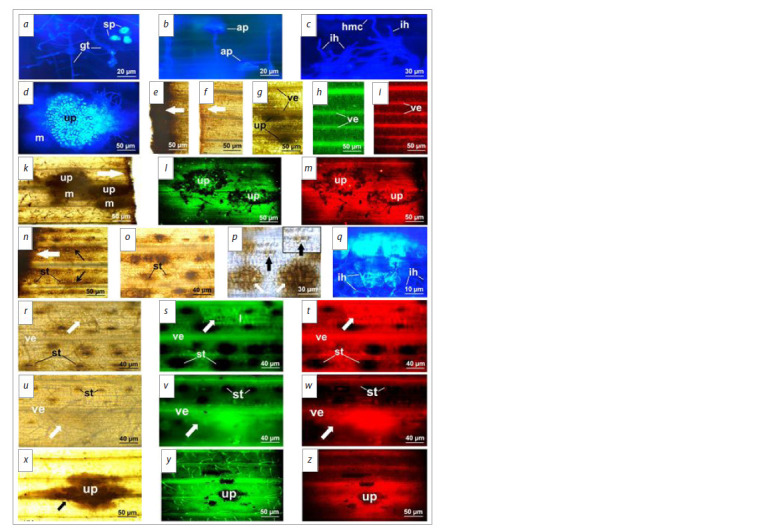
Development of P. graminis f. sp. tritici and distribution of hydrogen peroxide and phenols in tissues. a–d, g, k–m – infected untreated plants; e, f, h, i – control uninfected plants; n–z – Novochizol-treated infected plants. a – growing tubes development on the leaf surface; b – appressorium on stoma; c – infection hyphae and haustorial mother cell in the tissue; d – colony with urediniopustule; e – intensive H2O2 accumulation on the leaf cut of the control plant, 24 h p/in; f – weak H2O2 accumulation on the leaf section cut of the control plant, 240 h p/in; g – phenols in plant cytoplasm in the urediniopustule area and lignins in the parallel veins; h, i – phenols autofluorescence in the leaf of the control plant; k – H2O2 accumulation in the colony area with the urediniopustule and on the leaf cut (arrow), 240 h p/in; l, m – phenols autofluorescence in the tissues surrounding urediniopustules; n – intensive H2O2 accumulation on the leaf cut and in the plant tissue, 24 h p/in; o – H2O2 localization in the stomata area, 96 h p/in; p – empty appressorium shell on the plant stoma (black arrow, selected fragment) and intensive accumulation of H2O2 under other stomata (white arrows), 48 h p/ in; q – autofluorescence of dead plant cells and fungal infection hyphae, damaged cells (light blue) and normal hyphae (blue), 48 h p/ in; r – abortive colony (arrow); s, t – phenols and lignin autofluorescence in the same abortive colony (arrow) zone, 96 h p/in; u – actively developed colony (arrow); v, w – phenols autofluorescence in actively developed colony zone (arrows), 144 h p/ in; x – intensive H2O2 accumulation (arrow) in the colony zone with urediniopustule, 240 h p/in; y, z – intensive phenols accumulation with different colour illumination in the colony with urediniopustule zone, 240 h p/in. Designations: ap – appressorium; ih – infection hypha; l – lignin; hmc – haustorial mother cell; m – mycelium; ve – vein; gt – growing tube; sp – spore; st – stoma; up – urediniopustule. Staining: a–d, q – Uvitex 2B; e, f, k, n–p, r, u, x – DAB; g – aniline sulfate; h, l, s, v, y – phenols autofluorescence at emission λmax = 530 nm; i, m, t, w, z – phenols autofluorescence at emission λmax = 605 nm.

The Novochizol effects were assessed by Pgt development on the leaf surface and in the tissues, and plant reactions in the infection zone. After contact with the moistened plant surface, the urediniospores swelled and formed growing tubes (Fig. 1a). The appressoria were formed at the ends of most growing tubes, which were necessary for penetration into the stomata (Fig. 1b). A big part of the appressoria (73–78 %) were located on the stomata, and more than 93 % of them ensured pathogen penetration into the tissues. No significant differences in the development of Pgt on the surfaces of untreated and Novochizol-treated plants have been established (Table 2). The main appressoria proportion was formed 18–24 h p/in. After penetration into the stoma, the fungus formed infection hyphae with haustorial mother cells (Fig. 1c), and the first haustoria in mesophyll cells were formed 24–48 h p/in. Pgt formed large pustules with the next urediniospore generation 240 h p/in (Fig. 1d).

**Table 2.**
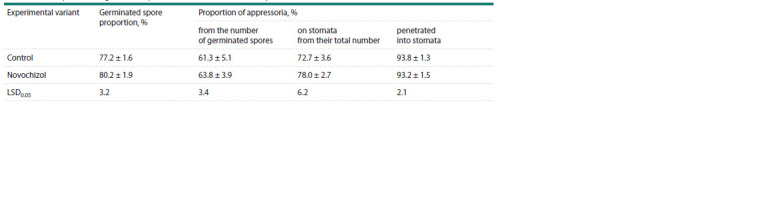
Development of P. graminis f. sp. tritici on the surface of wheat plants treated with Novochizol

The localization of hydrogen peroxide and phenolic compounds in the leaves was studied to determine active reactions. A high H2O2 concentration was revealed in leaf cuts by DAB staining at the beginning of the experiment in each variant (control untreated uninfected, Pgt-infected, Novochizoltreated, and Novochizol-treated infected plants) (Fig. 1e, n). In areas far from the cuts, DAB staining was weak (Fig. 1e). At the end of the experiment, DAB staining decreased significantly at the ends of all leaves (Fig. 1f, k). Probably, H2O2 generation at leaf ends was associated with plant stress reaction to mechanical damage.

The distribution of total phenols in the leaves was firstly studied using a special aniline sulphate staining. The phenols were detected in the cytoplasm and plant cell walls in the zone of Pgt development, as well as in the vein cell walls, which corresponds to the presence of polymer lignin. The phenols were low in other leaf parts (Fig. 1g). Phenol autofluorescence coincided with their localization, determined by aniline sulphate staining. Under different observation modes, a bright green or red fluorescence appeared (with emission at λmax = 530 nm or λmax = 605 nm, respectively). Different fluorescence colour is associated with the presence of different phenol compounds. In the control plants, red fluorescence was brighter in the midveins, and green fluorescence was more active in small veins, in particular, in the walls of stomatal guard and mesophyll cells (Fig. 1h, i).

In the untreated plants, significant changes in the cells in the infection zones were not found during pathogenesis, up to the sporulation stage. High H2O2 accumulation was determined in the tissues under the pustules, and less in the surrounding mycelium area at 240 h p/in (Fig. 1k). A moderate accumulation of phenols with green fluorescence and that of phenols with brighter red autofluorescence were detected around the pustules in the mycelium zones (Fig. 1l, m).

In the Novochizol-treated uninfected plants, H2O2 accumulation of varying intensity was noted in the leaves in the form of spots for 48 h p/in. The H2O2 distribution was irregular, which may be due to uneven Novochizol distribution by spraying. The stomatal guard cells, as well as mesophyll cells under the stomata and between the veins, were strongly stained (Fig. 1n). The H2O2 gradually disappeared from the tissues after 96–144 h p/in, but remained in the guard cells and in small zones below them in small amounts (Fig. 1o, r, u).

In Novochizol-treated infected plants, the H2O2 content in tissues for 96 hours was similar to that described above. The fungus penetrated into the stomata between H2O2 accumulation zones without deviations, and empty appressoria shells remained on the surface of guard cells (Fig. 1p). In areas with a high ROS content, the cytoplasm of dead plant cells showed a white glow, while damaged ones showed light blue fluorescence. The dead fungal hyphae had white autofluorescence, and the intact ones had a blue colour (Fig. 1q). The colonies died (aborted) at early developmental stages in the ROS accumulating loci. The phenols in the cytoplasm and lignin on the cell walls accumulated in the zones of the dead colonies. These substances had a brighter green and a less pronounced red autofluorescence after 96 h p/in. The accumulation of green and red lignins was also enhanced in the adjacent vein regions. At the same time, phenols did not accumulate near the stomata area with a high H2O2 content (Fig. 1s, t)

Significant H2O2 generation was not detected near the actively developing colonies 144 h p/in, and simultaneously its content decreased in the stomatal zones (Fig. 1u). Phenols with brighter green and less vivid red fluorescence covered the mycelium area (Fig. 1v, w). Intensive H2O2 accumulation was determined in the large colony and pustule areas 240 h p/ in (Fig. 1x), and strong phenol accumulation was noted around such colonies in a wider than H2O2 zone. Phenols and lignin with green autofluorescence were synthesized more intensively and spread over a larger area than ones with red colour (Fig. 1y, z).

**Fig. 2.**
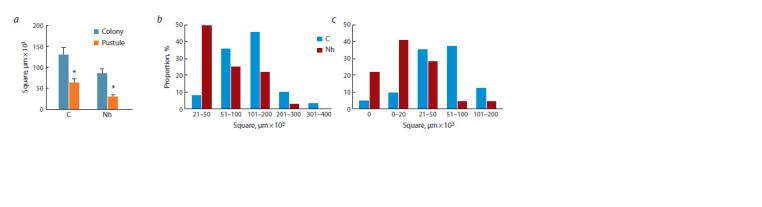
The effect of Novochizol treatment on P. graminis f. sp. tritici colonies and pustules development. a – сaverage area; b – distribution of colonies by area; c – distribution of pustules by area. С – control; Nh – Novochizol. * Significant difference at р ≤ 0.05.

A study of Pgt development showed that in Novochizoltreated plants, the average colony and pustule areas decreased (by 1.5 and 2.2 times to untreated, respectively) (Fig. 2a). The preparation’s effect resulted in a significant change in distribution of the colonies and pustules by sizes, compared with untreated plants. The proportion of small colonies and pustules increased sharply, and some colonies (22 %) died before sporulation (Fig. 2b, c).

## Discussion

The biological properties of the BPMA based on chitin and chitosan have been investigated since the 1980s. During this time, numerous tests have been carried out on the chitosan derivatives effects on pathogens (fungi, bacteria, and viruses) development and disease manifestations. The fungicidal effects of chitosans have been mainly studied on pathogens with a necrotrophic or hemibiotrophic feeding type. These groups include the most harmful species of the Botrytis, Fusarium, Alternaria, Colletotrichum, Phytophthora, Rhizoctonia genera (Chakraborty et al., 2020; Zheng et al., 2021; Shcherban, 2023). The cultivation of these fungi is available on artificial media, which makes it possible to evaluate the drug’s effects in vitro. Using different pathogen species, it was showed that the chitosan preparations manifested fungicidal effects by the suppression of spore germination, inhibition of growing tubes development, disruption of cell walls and membranes, and an impenetrable film formation around fungal cells (Ghaouth et al., 1994; Abd El-Kareem, Haggag, 2014). Growth suppression could also be associated with calcium and copper ions chelation and deposition of chelate complexes on cell surface, which reduced the metabolic activity of fungi (Chakraborty et al., 2020). The effects of preparations in planta are realized after chitosan recognition by plant receptors and signalling systems activation (Yarullina et al., 2023).

The mechanisms of Novoсhizol action on wheat stem rust development have been studied for the first time. The Novochizol concentration effect on the disease development has been revealed. This confirms the results of previous studies on the effect of drug doses on plant resistance reactions (Orzali et al., 2017; Varlamov et al., 2020). In the 0.75 % Novochizol variant, the greatest inhibiting effect on Pgt development was noted. In contrast to previous results, obtained with chitosan (Ghaouth et al., 1994; Abd El-Karee, Haggag, 2014), Novochizol had no negative effect on Pgt development on the leaf surface, as well as penetration into stomata.

In the 0.125 % Novochizol variant, a more intense pustule development suppression was found. The effect of the 0.125 % Novochizol on stem rust will be studied at the next research steps.

In the 2000s, a hypothesis of a two-level organization of plant immunity was formulated, called PTI-ETI (Gill et al., 2015). It was assumed that plants have PRRs (Pattern Recognition Receptors) that recognize molecules of non-pathogenic (MAMPs, Microbe-Associated Molecular Patterns) and non-specialized pathogenic microorganisms (PAMPs, Pathogen-Associated Molecular Patterns), as well as plant cell destruction products (DAMPs, Damage-Associated Molecular Patterns). As a result of the recognition of these molecules, the first level of PTI (PAMP-triggered immunity) defence is triggered. After PTI is overcome, the second resistance level is activated, associated with the recognition of specific effectors – ETI (Effector-Triggered Immunity). PTI corresponds to the response of non-host species, while ETI is similar to varietal resistance and is usually accompanied by hypersensitive reaction (Gill et al., 2015). Later, an improved model of plant immunity was proposed, according to which ETI is a PTI-dependent module for the reactions amplification, but not an isolated system (Yuan M. et al., 2021; Zhao et al., 2022).

Two peaks of ROS generation have been identified in resistant plants previously. The first peak occurs a few minutes after elicitor recognition and is associated with the activation of the NADP·H-oxidase enzyme, which is constitutively present in the membrane. NADP·H-oxidase produces the superoxide anion O •2–, which is rapidly converted by the SOD enzyme to H2O2 (Boller, Keen, 2000). The second ROS peak appears 3–5 days later, and is associated with de novo synthesis of the pro/antioxidant system enzymes (peroxidases, oxalate oxidases). The pro/antioxidant system maintains optimal ROS levels in the tissues. Catalase cleaves H2O2 to water, and at the same time the peroxidases, polyphenol oxidase, and ascorbate oxidase utilize ROS in oxidative reactions (Maksimov, Cherepanova, 2006).

Previously, when studying the interactions between the rust fungi P. triticina and P. coronata with non-host species (oats and wheat, respectively), the O •2– generation by stomatal guard cells contacting with appressoria, which led to pathogen death, was revealed (Plotnikova, 2008). Pgt dies on the plant surface before penetration into the stomata of non-host species Secale cereale and Thinopyrum ponticum. When Pgt interacts with cultivars carrying resistance genes of non-hosts (Sr31, Sr24, Sr25, Sr26), the appressoria dies on the stomata after the peak of superoxide anion generation (Plotnikova et al., 2022, 2023). On the example of chitosan-treated rice, a similar NADP·H-dependent O •2– synthesis was shown (Lopez-Moya et al., 2021). An enhanced synthesis of enzymes involved in ROS accumulation was also found in millet plants treated with chitosan and infected with Alternaria kikuchiana (Meng et al., 2010). The chitosan application on barley induces an oxidative burst and synthesis of phenolic compounds, which increases the resistance to fungal diseases complex (Faoro et al., 2008).

Novoсhizol is similar to MAMPs in its origin. A histochemical study of Novochizol-treated plants revealed intensive H2O2 accumulation in tissues four days after its application. Obviously, this is due to the second peak of oxidative burst manifestation and confirms the inducing resistance activity of Novochizol. Zones with a high H2O2 content were found both in the stomata areas and between the veins. Such results may be explained by increased Novochizol ability to penetrate through the leaf epidermis and induce ROS production. The H2O2 content in the tissues decreased after 96–144 h p/in of Pgt inoculation, so did the traumatic ROS on leaf cuts to the end of the experiment in all variants. Such dynamics may be related to the synthesis of the antioxidant system components (both the enzymes and non-enzymatic substances) that utilize ROS. The activation of antioxidant enzymes following ROS accumulation was shown in potatoes treated with the chitinferulic acid conjugate and beneficial bacteria Bacillus subtilis (Yarullina et al., 2024a). It is also possible that the antioxidant activity increased with the age of the plants.

Defense reactions did not appeared before sporogenesis in infected untreated plants. In Novochizol-treated plants, a small number of host cells and mycelium fragments died in the areas with increased H2O2 content. At the same time, the dead plant cells did not exhibit the yellow fluorescence characteristic for HR (Vander et al., 1998). This indicates that Novochizol induces reactions that partially differ from those occurring during HR in resistant varieties.

Some colonies died at the early pathogenesis stages. H2O2 was not detected in the zones of abortive colonies 96 h p/ in. ROS accumulation has also not been established in the areas of medium and large developing colonies before sporogenesis, and even a decrease in H2O2 near the colonies has been noted. The decrease in H2O2 content can be explained both by the accumulation of antioxidant plant enzymes and by the pathogen activity. Currently, it is known that biotrophic rust fungi secrete hundreds of effectors into plant cytoplasm and apoplast. The pathogens are able to suppress protective reactions, as well as to alter or reprogram host metabolism by the effectors. It was shown that a virulent isolate of wheat yellow rust pathogen P. striiformis f. sp. tritici secreted an effector catalase cleaving H2O2, which led to plant resistance suppression (Yuan P. et al., 2021). At the same time, Novochizol treatment stimulated increasing H2O2 accumulation in the colony zones at the stage of sporogenesis.

It has previously been shown that the phenols synthesis and the strengthening of cell walls with lignins after treatment with chitosans were the most typical protective reactions against necrotrophic and hemibiotrophic fungi (Orzali et al., 2017; Shcherban, 2023). In our experiments, it was found that Novochizol treatment stimulated an earlier and more intensive phenols accumulation than in untreated plants. For the first time, it was shown that Novochizol promotes the changing in the phenols ratio towards compounds with a green fluorescence, while phenols with a red light prevailed in untreated plants. It was previously determined that lignin with green autofluorescence includes syringin derivatives and accumulates in wheat tissues after treatment with SAR inducer Bion (Plotnikova, 2009). Previously, it was shown that in plants treated with the chitosans and infected with necrotrophic fungi, the PR proteins’ (chitinases, glucanases, peroxidases, polyphenol oxidases, PR-1, PR-5, etc.) genes expression increased (Manjunatha et al., 2008, 2009; Nandeeshkumar et al., 2008; Orzali et al., 2014). Similar accumulation of PR proteins with different functions was also revealed in potatoes treated by chitosan conjugates with ferulic or caffeic acids (Yarullina et al., 2024a, b). Accumulation of PR-proteins in Novochizol-treated plants, which are not detectable by cytological methods, is also likely. The complex action of Novochizol-induced defence mechanisms led to the death of a significant part of the colonies at the early development stages, as well as to a significant reduction in the pustule density and suppression of the pathogen’s reproduction

The reported studies were the first stage of investigation of the Novochizol effect on the wheat resistance mechanisms against stem rust. At the next stage, detailed studies of the preparation’s action on the pathogenesis will be carried out using molecular genetics, biochemical and cytophysiological methods.

## Conclusion

Studies have shown that Novohizol can be used as a resistance inducer to wheat stem rust. The dose effect of the treatment was revealed, with the best results at 0.125 and 0.75 % concentrations

Novochizol treatment of leaves at the 0.75 % concentration did not affect the urediniospore germination and fungal structures development on the plant surface, but led to a significant reduction in the number of colonies, as well as the mycelium and pustule sizes.

Intensive hydrogen peroxide accumulation in infected and uninfected plant tissues 4–8 days after Novochizol treatment was found (corresponds to 0–4 days after inoculation), which decreased by the end of the experiment

Partial death of plant cells and pathogen mycelium was noted in the zones of intensive H2O2 accumulation. The dead plant cells did not show the autofluorescence characteristic for HR.

Novochizol stimulated earlier and more intensive phenols accumulation in infection zones, such as a change in the ratio of phenolic compounds towards substances with syringin derivatives.

## Conflict of interest

The authors declare no conflict of interest.
